# Secondary Structure Preferences of Mn^**2+**^ Binding Sites in Bacterial Proteins

**DOI:** 10.1155/2014/501841

**Published:** 2014-03-17

**Authors:** Tatyana Aleksandrovna Khrustaleva

**Affiliations:** Regulatory Proteins and Peptides Laboratory, Institute of Physiology of the National Academy of Sciences of Belarus, Akademicheskaya 28, 220072 Minsk, Belarus

## Abstract

3D structures of proteins with coordinated Mn^2+^ ions from bacteria with low, average, and high genomic GC-content have been analyzed (149 PDB files were used). Major Mn^2+^ binders are aspartic acid (6.82% of Asp residues), histidine (14.76% of His residues), and glutamic acid (3.51% of Glu residues). We found out that the motif of secondary structure “beta strand-major binder-random coil” is overrepresented around all the three major Mn^2+^ binders. That motif may be followed by either alpha helix or beta strand. Beta strands near Mn^2+^ binding residues should be stable because they are enriched by such beta formers as valine and isoleucine, as well as by specific combinations of hydrophobic and hydrophilic amino acid residues characteristic to beta sheet. In the group of proteins from GC-rich bacteria glutamic acid residues situated in alpha helices frequently coordinate Mn^2+^ ions, probably, because of the decrease of Lys usage under the influence of mutational GC-pressure. On the other hand, the percentage of Mn^2+^ sites with at least one amino acid in the “beta strand-major binder-random coil” motif of secondary structure (77.88%) does not depend on genomic GC-content.

## 1. Introduction

In general, there are three “major binders” of Mn^2+^ ions: oxygen atoms from carboxyl groups of aspartic and glutamic acids side chains and imidazole nitrogen atom from histidine side chain [1, 2]. Minor binders are oxygen atoms from hydroxyl groups of serine and threonine side chains; amide nitrogen and oxygen atoms from asparagine and glutamine side chains; sulfur atoms from thiol group of cysteine and thioether group of methionine; and oxygen atoms from peptide bonds of all the amino acids including even hydrophobic ones [1, 2].

There is some controversy in the results of* in silico* studies on amino acid preferences for Mn^2+^ binding. According to the work of Zheng et al. [1], there are three amino acid residues most frequently found in Mn^2+^ binding sites: His, Asp, and Glu. Histidine has the highest normalized frequency in binding sites, while glutamic acid has the lowest normalized frequency among those three amino acid residues [1]. According to the work of Brylinski and Skolnick [2], aspartic acid has much higher preference to bind Mn^2+^ than glutamic acid and histidine.

Information on amino acid preferences and geometry of coordination spheres is used in algorithms for metal binding sites prediction, such as FINDSITE-metal [2], MetalDetector v2.0 [3], Fold-X [4], and FlexX [5]. However, the information on preferable 3D structural motifs is available mostly for Ca^2+^ and Zn^2+^ binding proteins. Well-known EF-hand motif for Ca^2+^ binding consists of two alpha helices and a loop between them [6]. The first helix known as E consists of 10–12 residues, and the second helix known as F also consists of 10–12 residues. The angle between those helices is close to 90°. The loop between the helices approximately 12 residues in length often includes “Asp-Xaa-Asp-Xaa-Asp-Gly” motif which is directly involved in Ca^2+^ coordination [7]. Recently, other proteins, able to bind Ca^2+^ containing the abovementioned motif but lacking one or both helices, have been described [8]. As to Zn^2+^ binding 3D structural motifs, Sri Krishna et al. classified them in eight different groups.

The aim of this study was to find out whether there is a secondary structural motif which is characteristic for relatively short parts of polypeptide chains around Mn^2+^ binding amino acid residues.

In fact, the same kind of secondary structural motif may be found in several 3D structural motifs. For example, four from eight 3D structural motifs for Zn^2+^ binding include such a secondary structural motif as beta hairpin. That is why the knowledge on preferable secondary structural motifs around each of the amino acid residues may be even more helpful for prediction of ion binding sites than the knowledge on the 3D structural motifs for the complete coordination spheres. Amino acid preferences have also been studied in the present work not just for binding residues but also for their neighbors.

It is known that amino acid content is not constant among proteins. The major cause of variations in amino acid content is symmetric mutational pressure [9]. Frequencies of those amino acid residues in proteomes which are encoded by GC-rich codons (Ala, Gly, Pro, and Arg) show direct dependence on GC-content of genomes [10]. The slope of that dependence for alanine is the steepest one [11]. Frequencies of those amino acid residues in proteomes which are encoded by GC-poor codons (Ile, Lys, Asn, Phe, Tyr, and Met) show inverse dependence on GC-content of genomes [10]. Slopes for isoleucine, lysine, and asparagine are steeper than those for phenylalanine, tyrosine, and methionine [12].

It is known that tertiary and secondary structures are more conserved in proteins than their primary sequences. That phenomenon is known as protein structure degeneracy. Different amino acid residues may substitute each other, while secondary and tertiary structures stay almost the same for homologous proteins because of the negative selection [13]. One may predict that secondary structure distribution around the most of residues binding the same cation will be similar for proteins with different amino acid content. However, that statement has to be tested in each particular study.

Even though three amino acids most frequently involved in Mn^2+^ binding (Asp, Glu, and His) are encoded by codons of average GC-content, their binding features and patterns of secondary structure distribution around them may depend on GC-content of genes. There are some interesting consequences of the growth of genomic GC-content which may bring some changes into the structure of Mn^2+^ binding sites. For example, total levels of both strongly hydrophobic and strongly hydrophilic amino acids in proteins show inverse dependence on G+C [11, 13]. The usage of sheet-like pentapeptides grows in alpha helices and in random coil due to mutational GC-pressure [14]. That is why we decided to study Mn^2+^ binding sites in three groups of bacterial proteins: from bacterial species with low, average, and high genomic GC-content. The same kind of methodology may be used in studies on other properties of proteins. Changes in amino acid content that occurred due to symmetric mutational pressure may theoretically result in reorganization of binding sites for certain ligands or even in the availability of potential binding sites.

## 2. Materials and Methods

Three sets of PDB files containing Mn^2+^ ions coordinated by amino acid residues have been collected from the Protein Data Bank (http://www.pdb.org). The total number of those files was equal to 149. The first set includes 39 PDB files with 3D structures of proteins from bacteria with genomic GC-content lower than 40%. The second set includes 62 PDB files with 3D structures of proteins from bacteria with average genomic GC-content (from 40% to 60%). The third set is composed of 48 PDB files with 3D structures of proteins from bacteria with GC-rich genomes (G + C > 60%). Identical proteins have not been used in this study, as well as close homologues. According to the results of the “decrease redundancy” algorithm (http://web.expasy.org/decrease_redundancy/), there were no sequences with similarity level higher than 60% in each of the three data sets.

GC-poor bacteria used in this study are* Bacillus subtilis* (18 files);* Bacillus anthracis* (4 files);* Bacillus caldovelox* (1 file);* Bacillus cereus* (1 file);* Clostridium cellulolyticum* (1 file);* Haemophilus influenzae* (3 files);* Listeria monocytogenes* (2 files);* Staphylococcus aureus* (5 files); and* Streptococcus pneumoniae* (4 files).

Bacteria with average genomic GC-content are* Escherichia coli* (38 files);* Brucella melitensis* (2 files);* Geobacillus stearothermophilus* (4 files);* Neisseria meningitidis* (3 files);* Paenibacillus polymyxa* (1 file);* Salmonella typhimurium* (1 file);* Synechocystis *sp. (4 files); and* Thermotoga maritima* (9 files).

The list of bacteria with GC-rich genomes is the following:* Mycobacterium tuberculosis* (19 files);* Deinococcus radiodurans* (4 files);* Pseudomonas aeruginosa* (5 files);* Pseudomonas cichorii* (1 file);* Pseudomonas putida* (3 files);* Pseudomonas stutzeri* (2 files);* Streptomyces rubiginosus* (1 file);* Thermus thermophilus* (10 files); and* Xanthomonas campestris* (3 files).

Complete list of PDB identifiers can be found in the supplementary material file “PDB identifiers.xlsx;” (see Supplementary Material available online at http://dx.doi.org/10.1155/2014/501841). The data on classification displayed in “Annotations” section of PDB pages were available for almost one half of proteins. About 54% of proteins were classified according to CATH (Class, Architecture, Topology, Homologous superfamily), and 49% were classified according to SCOP (Structural Classification of Proteins). From all proteins classified according to CATH, 77% were alpha and beta proteins, 7% were mostly alpha proteins, and 5% were mostly beta ones, while 11% of them contained several different domains. From all proteins classified by SCOP, 47% were alpha and beta (a/b) proteins, 16% were alpha and beta (a + b) proteins, 10% were* all* alpha proteins, and 4% were* all* beta proteins, while 23% of them were mixed proteins. So, most of the studied proteins contain both alpha helices and beta strands. Percentage of parallel beta strands is higher than that of antiparallel beta strands. It is also important to mention that 85% of proteins used in this study are enzymes. Most of the Mn^2+^ coordinating sites should be involved in enzymatic activity.

We used descriptions of Mn^2+^ binding sites which can be found in PDB files. For each of the amino acid residues involved in Mn^2+^ coordination, the following data have been collected: (i) amino acid residues situated in five positions towards N-terminus (−5/−4/−3/−2/−1) and C-terminus (+1/+2/+3/+4/+5) from the binding residue; (ii) secondary structure of those amino acid residues and of the binding residue itself. In other words, we collected three sets of short amino acid sequences (11 amino acids in length) with the Mn^2+^ binding residue in the center of each of them.

Certain amino acid residues may be included in two binding sites (for different Mn^2+^ ions). To avoid the bias in our data set, we deleted repeated records. Finally, there were 161 amino acids involved in Mn^2+^ binding in proteins from GC-poor bacteria; 248 amino acids in proteins from bacteria with average genomic G + C; and 194 amino acids in proteins from GC-rich bacteria.

There are three amino acid residues (major binders) most frequently coordinating Mn^2+^ ions: aspartic acid, histidine, and glutamic acid. We repeated the procedure of data extraction for Asp, His, and Glu residues which* are not involved* in Mn^2+^ binding in the common set of PDB files. There were 2813 Asp, 1080 His, and 3572 Glu residues in the “control” data set.

Three sets of amino acid sequences containing Mn^2+^ binding residues in their centers are available in supplementary material file “Mn(II) binding sites.xlsx”. Three control sets of amino acid sequences with those major binders (Asp, His, and Glu) which did not coordinate Mn^2+^ in their centers can be found in supplementary material file “D, H and E residues non binding Mn(II).xlsx”.

Amino acid usage in each of the ten positions around each of the three major binders has been calculated for binding and nonbinding residues. Then, probabilities to be situated around each of the major binders have been calculated as ratios between the usage of a given amino acid in the certain position near the binding residue and the sum of its usages around binding and nonbinding residues. Statistical significance of those probabilities has been acquired from the results of two-tailed *t*-test. Similar statistical procedure has been performed for secondary structure elements around binding and nonbinding Asp, His, and Glu residues.

For calculation of amino acid frequencies in proteins from three data sets, we deleted their polyhistidine tails. This procedure was important for correct calculation of the percentage of His residues involved in Mn^2+^ binding. We also calculated percentage of Asp and Glu residues involved in Mn^2+^ coordination (relatively to their total usages).

Average usages of Lys and Arg have been calculated near binding and nonbinding glutamic acid residues being in alpha helix, beta strand, and random coil.

To complete analyses of secondary structure motifs involved in Mn^2+^ coordination, we compared by* t*-test usages of amino acids situated in certain types of secondary and supersecondary structure in the set of binding residues and in the whole set of amino acids. For this* in silico* experiment, we used alpha helices, beta strands, four types of coil regions (BCH: coil between beta strand and alpha helix; HCB: coil between alpha helix and beta strand; BCB: coil between two beta strands; and HCH: coil between two alpha helices), and four types of supersecondary structural motifs (B-BCH-H: beta strand and alpha helix separated by a region of coil; H-HCB-B: alpha helix and beta strand separated by the region of coil; B-BCB-B: two beta strands and coil between them; H-HCH-H: two alpha helices and coil between them).

There are 15 apo forms available in the Protein Data Bank for proteins used in this study. Amino acid sequences of apo and holo forms are 100% identical. All other ligands, except Mn^2+^ cation(s), are identical for apo and holo forms. Secondary structures around Mn^2+^ coordinating residues have been compared for holo and apo forms in subsequent pairs: 1VHA-1VH8; 3F8N-2RGV; 1ONO-1ONN; 1WSE-1WSH; 1XUU-3CM4; 2D0C-2D0A; 2HXG-2AJT; 2NRZ-2NRW; 3GME-3GLL; 3TMY-1TMY; 2E6C-2E69; 2YES-2WE9; 2YF3-2YF4; 2ZXP-2ZXO; 3ITX-3ITY.

Types of pentapeptides composed of hydrophilic and hydrophobic amino acids have been determined for “−5–−1” and “−4–0” positions for Asp, Glu, and His residues. Amino acid residues have been classified into hydrophilic (W) and hydrophobic (O) ones according to the Eisenberg scale [15] in which Asp, Glu, His, Gln, Ser, Thr, Arg, Asn, and Lys are hydrophilic. Percentages of sheet-like pentapeptides [14] in beta strands situated in the N-terminal direction from the binding and nonbinding Asp, Glu, and His residues have been compared by *t*-test.

## 3. Results

### 3.1. Amino Acids Involved in Mn^2+^ Binding

The percentage of aspartic acid residues in Mn^2+^ binding sites is equal to 34.16%. The percentage of histidine residues in those sites is somewhat lower (31.01%), while the difference between them is not significant (*P* > 0.05). The percentage of glutamic acid residues in Mn^2+^ binding sites (21.56%) is significantly lower than those for aspartic acid and histidine (*P* < 0.001). As one can see in Table 1, this situation is characteristic for all the three groups of proteins. There is no dependence between GC-content of genes and the distribution of three major Mn^2+^ binders (aspartic acid, histidine, and glutamic acid) in binding sites.

On the other hand, the difference between the usage of all other amino acid residues (minor binders) in those sites from proteins encoded by GC-rich genes (6.70%) and proteins encoded by genes with average GC-content (17.34%) is significant (*P* < 0.001). The difference between the sum of minor Mn^2+^ binders for proteins encoded by GC-rich and GC-poor genes is also significant (6.70% versus 14.91%; *P* < 0.01). This fact can be explained by the known tendency: total usage of hydrophilic amino acid residues in proteins decreases with the growth of GC-content in genes [11, 13].

It is also important to calculate the percentage of amino acid residues involved in Mn^2+^ binding relative to their average usage in proteins. It is known that histidine is one of the rare amino acids, while glutamic acid is even more abundant than aspartic acid [10, 12]. In the proteins from our data set, amino acid usages of the major Mn^2+^ binders are as follows: Glu: 7.59 ± 0.34%; Asp: 6.22 ± 0.24%; His: 2.68 ± 0.19%. One can easily come to the conclusion that histidine is overrepresented in Mn^2+^ binding sites relatively to aspartic and, especially, glutamic acids. Indeed, 14.76% of histidine residues are involved in Mn^2+^ binding. In contrast, 6.82% of aspartic and just 3.51% of glutamic acid residues participate in binding of that ion (ions). GC-content of genes does not significantly influence the percentage of His, Asp, and Glu residues involved in Mn^2+^ binding by proteins (see Table 1).

It is important to mention that 17.69% of glutamic acid residues and 13.59% of aspartic acid residues participated in binding of two Mn^2+^ ions. Histidine residues cannot bind two Mn^2+^ ions simultaneously.

So, the major Mn^2+^ binders are Asp, His, and Glu. His is overrepresented in Mn^2+^ binding sites relatively to Asp, while Glu is underrepresented.

### 3.2. Secondary Structure of the Region around the Aspartic Acid Involved in Mn^2+^ Binding

We compared distribution of secondary structure elements around the Asp residues involved in Mn^2+^ binding and those Asp residues which are not involved in that ion coordination. Probabilities for Asp to be Mn^2+^ binding residue are given in Table 2. As one can see in Table 2, beta strand is significantly overrepresented in −5, −4, −3, −2, −1, 0, and +1 positions from the Asp residues which bind Mn^2+^ relatively to those which do not bind that ion. It means that there is usually a beta strand near the Asp from Mn^2+^ binding sites. Interestingly, that beta strand can usually be found in the N-terminal direction and not in the C-terminal one. Random coil is significantly overrepresented in +1, +2, +3, +4, and +5 positions (see Table 2). So, Asp residues binding Mn^2+^ are usually surrounded by the beta strand in the N-terminal direction and random coil in the C-terminal direction. Alpha helix and helix 3/10 are, in general, underrepresented around Asp residues involved in Mn^2+^ coordination.

Most of the preferences in amino acid distribution near Asp residues binding Mn^2+^ can be explained by their secondary structure formation propensities. Such strong beta strand formers as valine and isoleucine [16] are overrepresented in certain positions in the N-terminal direction from the Asp residues binding Mn^2+^. Even though leucine is usually described as strong helix former [16], it is often involved in beta strand formation because of its hydrophobicity [14]. That is why leucine is significantly overrepresented in −5, −4, and −3 positions (see Table 2). Three other strong helix formers (Ala, Glu, and Gln) are underrepresented in certain positions in the N-terminal direction from the Asp involved in Mn^2+^ binding (see Table 2). As to Arg and Lys, which are listed among helix formers too [16], their underrepresentation can be linked with the positive charge of their side chains as well.

It is important to highlight that Asp residues are significantly overrepresented in −2 and +2 positions around the Asp residues binding Mn^2+^. One may think that there should be many Asp-Xaa-Asp-Xaa-Asp-Gly motifs in Mn^2+^ binding sites; see Table 2. However, this type of site characteristic for Ca^2+^ binding regions [17] was found only once in our data set. There are also just two Asp-Xaa-Asp-Xaa-Xaa-Gly and three Xaa-Xaa-Asp-Xaa-Asp-Gly sites which are similar to canonical sites for Ca^2+^ binding. So, relatively short Asp-Xaa-Asp and Asp-Xaa-Xaa-Gly motifs seem to be characteristic for Mn^2+^ binding sites. Histidine residues are also overrepresented around Asp interacting with Mn^2+^ (in −2, +1, +2, +3, and +4 positions). Serine which may sometimes provide its –OH group for Mn^2+^ coordination is overrepresented in −1 position, while threonine also possessing that kind of group is overrepresented in +1 position. Asparagine with carboxamide group able to participate in Mn^2+^ coordination can frequently be found in +2 position (see Table 2). From these data, we can conclude that Mn^2+^ binders can often be found in the same linear sequence. Minor binders (such as Ser, Thr, and Asn) are involved in binding mostly in case if they are close neighbors of the major binders. On the other hand, they can contribute to the total hydrophilicity of the binding area.

Glycine is overrepresented in −5, −1, and +3 positions probably contributing into the flexibility of the Asp residue involved in Mn^2+^ binding. Being a strong secondary structure breaker [16], proline is underrepresented in −3, −1, +1, and +2 positions, while it is overrepresented in +4 position.

In general, Mn^2+^ binding aspartic acid residue is usually surrounded by hydrophobic amino acids (Val, Ile, and Leu) which form beta strand in the N-terminal direction and coil formers (His, Asp, Asn, Pro, and Gly) in the C-terminal direction. Major (His, Asp) and minor (Ser, Thr, and Asn) Mn^2+^ binders are overrepresented near that residue.

**Figure 1:**
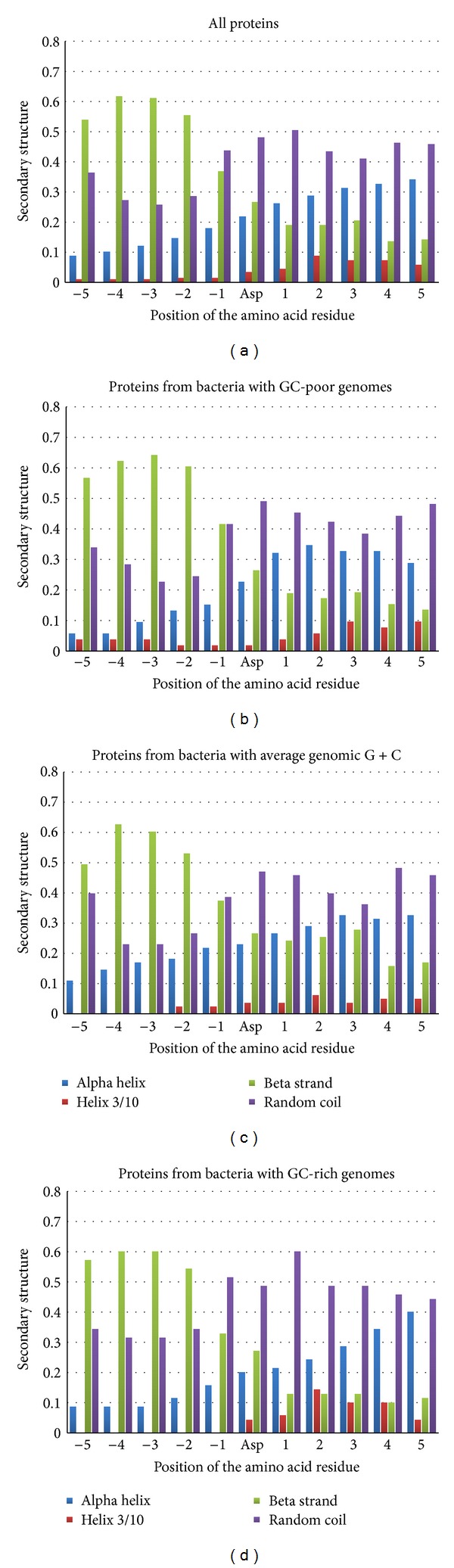
Secondary structure distribution around aspartic acid residues binding Mn^+2^ ions in all the proteins (a); in proteins from GC-poor bacteria (b); in proteins from bacteria with average genomic GC-content (c); in proteins from GC-rich bacteria (d).

In Figure 1, one can see the concrete distribution of secondary structure elements around Asp residues binding Mn^2+^. More than 60% of amino acid residues in −4 and −3 positions form beta strand (see Figure 1(a)). The percentage of amino acid residues forming beta strand is also high in −5 and −2 positions (see Figure 1(a)). This preference for beta strand from −5 to −2 positions is characteristic for proteins encoded by GC-poor (Figure 1(b)) and GC-rich genes (Figure 1(d)), as well as for proteins encoded by genes with average GC-content (Figure 1(c)).

Random coil is the most frequently observed conformation of amino acid residues near the aspartic acid involved in Mn^2+^ binding in the positions from −1 to +5. This tendency is characteristic for all the three groups of proteins encoded by genes of different GC-content (see Figure 1).

Secondary structure near Asp residues which are not involved in Mn^2+^ binding is quite different from that represented in Figure 1. Alpha helix is the most frequently observed element of secondary structure from −5 to −3 and from +1 to +5 positions (about 35–45%). Random coil is most frequently observed from −2 to 0 positions only (about 40–45%). Beta strand can rarely be found near the Asp residue which is not involved in Mn^2+^ binding. The highest frequency is characteristic to −5 and +5 positions (above 20%).

There is a clear preference for asymmetric secondary structure distribution around aspartic acid residues providing oxygen atoms from their side chains for Mn^2+^ coordination: beta strand is situated in the N-terminal direction, while random coil is situated in the C-terminal direction.

### 3.3. Secondary Structure of the Region around the Histidine Involved in Mn^2+^ Binding

Preferable secondary structure around histidine residues binding Mn^2+^ (see Table 3) is similar to that around aspartic acid residues. Beta strand is the preferable type of secondary structure for positions from −5 to 0. Random coil is overrepresented from +2 to +5 positions (see Table 3). Alpha helix is underrepresented around histidine residues binding Mn^2+^.

Amino acid preferences for ten positions near Mn^2+^ binding histidine residues do not have too much in common with those near aspartic acid residues (see Table 3). The only one overrepresented beta strand former is isoleucine (in −5 and −4 positions). Interestingly, alanine (strong helix former) has some position specific preferences: it is underrepresented in −3, −1, and +5 positions, but it is overrepresented in +1 position (see Table 3). Glycine is overrepresented in −3 position, while proline is underrepresented in −2 and +1 positions.

Major Mn^2+^ binders are grouped in the following way: His in −2 and +2 positions; Asp in −1 position; Glu in +5 position. As to the minor Mn^2+^ binders, Ser is overrepresented in +1 position; Asn is overrepresented in +4 position, and Thr is overrepresented in +2 and +5 positions.

Positively charged arginine is underrepresented in −2 and +1 positions, while lysine is significantly underrepresented in −3, −2, −1, +2, and +4 positions.

**Figure 2:**
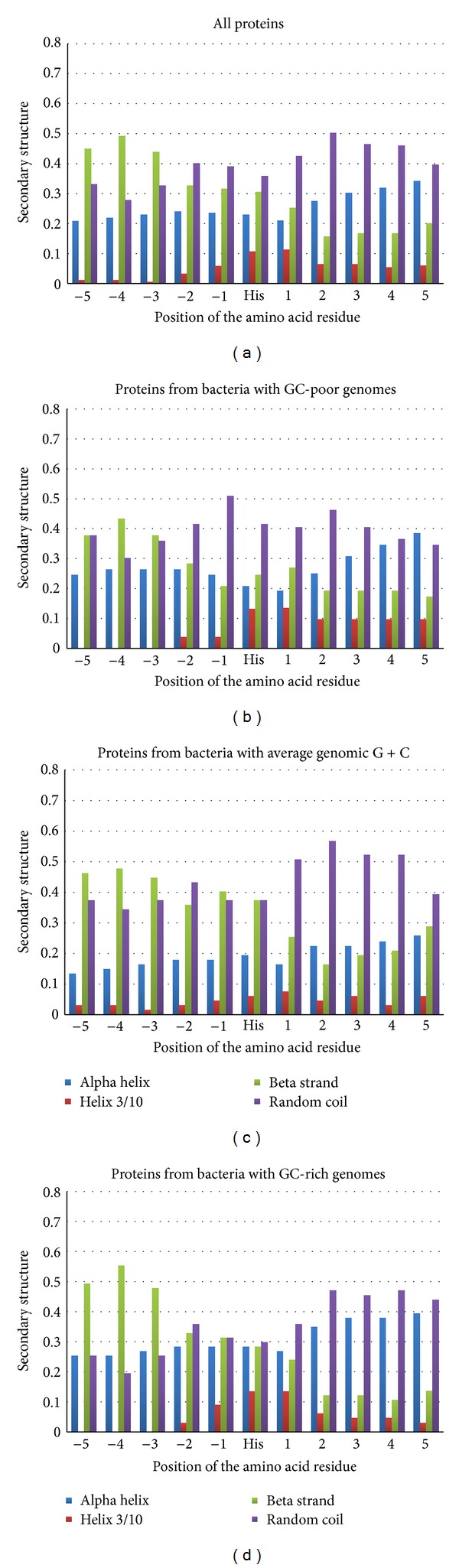
Secondary structure distribution around histidine residues binding Mn^+2^ ions in all the proteins (a); in proteins from GC-poor bacteria (b); in proteins from bacteria with average genomic GC-content (c); in proteins from GC-rich bacteria (d).

In Figure 2(a), one can see that beta strand is the preferable conformation for amino acid residues from −5 to −3 positions. However, frequencies of amino acid residues in beta strand conformation in those positions are somewhat lower for histidine surroundings (about 45%) than for aspartic acid surroundings. Random coil is the favorable conformation from −2 to +5 positions. There are some variations on this common theme in Figures 2(b)–2(d), while in general GC-content of genes seems to have no influence on the preferable secondary structure around histidine residues binding Mn^2+^.

Secondary structure elements around histidine residues not involved in Mn^2+^ binding are distributed in the following way: alpha helix is preferable (from 35 to 45%) for all positions, except −1 position with the preference for random coil; the difference between percentage of helix and percentage of coil is low; the percentage of beta strand in all positions is close to 20%.

Manganese (II) ions binding histidine residues are usually surrounded by the same kind of asymmetric secondary structure elements as aspartic acid residues.

### 3.4. Secondary Structure of the Region around the Glutamic Acid Involved in Mn^2+^ Binding

There is a clear preference for beta strand situated from −4 to +2 positions for glutamic acid residues involved in Mn^2+^ binding (see Table 4). Random coil is overrepresented in +5 position only (see Table 4). These data confirm that “beta strand-major binder-random coil” secondary structural motif is a characteristic of all the three major Mn^2+^ binders (Asp, His, and Glu).

Hydrophobic amino acids known as strong beta strand formers are overrepresented in the N-terminal direction from the Glu residues binding Mn^2+^ (see Table 4). Valine is overrepresented in −3 and +1 positions; isoleucine is overrepresented in −1 position; phenylalanine is overrepresented in −3 position.

Among major and minor Mn^2+^ binders, only histidine is significantly overrepresented in +3 position (see Table 4). Once again, arginine is underrepresented in three positions, while lysine is underrepresented in five different positions (see Table 4).

**Figure 3:**
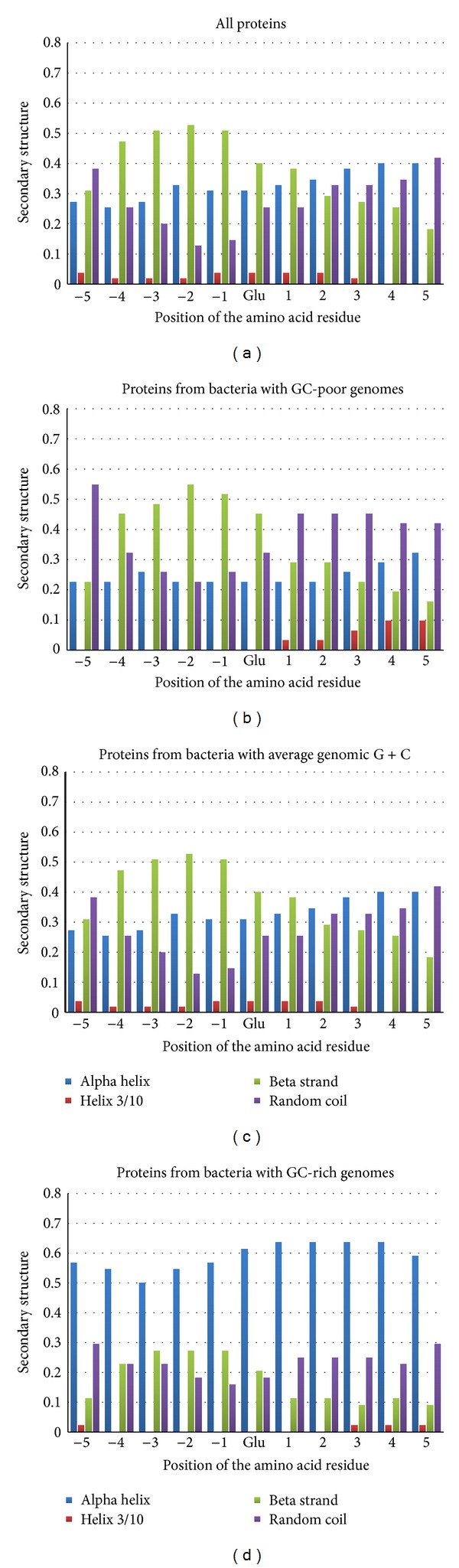
Secondary structure distribution around glutamic acid residues binding Mn^+2^ ions in all the proteins (a); in proteins from GC-poor bacteria (b); in proteins from bacteria with average genomic GC-content (c); in proteins from GC-rich bacteria (d).

In proteins encoded by GC-poor genes and by genes with average G + C, the pattern of secondary structure distribution around Glu residues binding Mn^2+^ (see Figures 3(b) and 3(c)) is in general similar to the patterns found around aspartic acid and histidine. However, in proteins encoded by GC-rich genes, glutamic acid preferably binds Mn^2+^ being included in alpha helix (see Figure 3(d)). The kind of secondary structure elements distribution shown in Figure 3(d) is similar to that for Glu residues which do not bind Mn^2+^ (percentage of alpha helix is about 45–50% in all positions, percentage of coil is equal to approximately 30%, and the rest is left for beta strand and helix 3/10). However, the traces of the preference for beta strand from −4 to 0 positions still can be seen in Figure 3(d).

Even though the most commonly distributed kind of secondary structural motif (beta strand-major binder-random coil) is characteristic for Glu residues binding Mn^2+^, in proteins encoded by GC-rich genes glutamic acid residues from alpha helices became able to bind that ion too.

### 3.5. Secondary Structures in Mn^2+^ Coordinating Spheres without “Beta Strand-Major Binder-Random Coil” Motif

The number of Mn^2+^ coordinating spheres which contain at least one binding residue situated in the “beta strand-major binder-random coil” motif is equal to 77.8%. Coordinating sites without that motif demonstrate some characteristic features. The most frequently used binder in those sites is Glu (51.6%). Two other major binders (Asp, 15.6%, and His, 23.0%) are used less frequently, while the percentage of all other amino acids participating in Mn^2+^ coordination is relatively high (25.4%).

Secondary structure distribution around Glu residues from the described type of Mn^2+^ binding sites is very specific: alpha helix is found in 80–90% of cases in all the positions around glutamic acid. Lysine residues are underrepresented in −4, −3, −2, −1, +1, and +3 positions around Glu, while arginine residues are underrepresented in −3 and +3 positions and overrepresented in −2 and +2 positions. It means that Arg situated on the different surface of alpha helix cannot disturb Mn^2+^ binding by Glu, unlike Arg situated on the same surface. As to Lys, its high frequency in helices seems to be the main cause of their low level of usage around Mn^2+^ coordinating residues. However, those helices (or regions of helices) which have no lysine residues are able to bind Mn^2+^.

Some parts of coordination spheres which do not have any binder that fit within the dominant pattern (29.2%) contain just a single amino acid residue coordinating Mn^2+^ cation. Other ligands included in coordination spheres together with those single amino acid residues should be responsible for the cation binding.

### 3.6. Decrease of Lysine Usage as the Most Probable Cause of the GC-Pressure Induced Switch in Structural Types of Mn^2+^ Binding Sites for Glutamic Acid

As one can see in Tables 2–4, lysine is underrepresented around Asp, His, and Glu residues binding Mn^2+^ much more than any other amino acid. Lysine is encoded by GC-poor codons (AAA and AAG). It is known that total level of lysine usage in proteins decreases steeply with the growth of G + C in genes [12]. Indeed, in the set of proteins used in the present work, the usage of lysine is equal to 7.21 ± 0.66% for proteins from GC-poor bacteria; 5.94 ± 0.58% for proteins from bacteria with average genomic G + C; and just 2.92 ± 0.55% for proteins from GC-rich bacteria.

**Figure 4:**
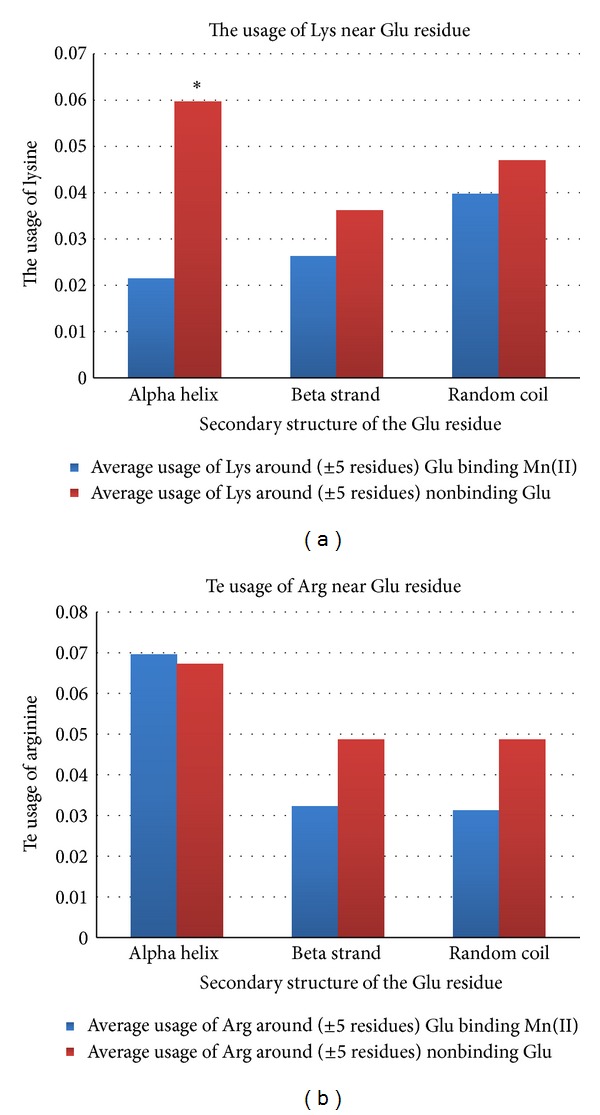
The usage of lysine (a) and arginine (b) around glutamic acid residues binding Mn^+2^ and nonbinding ones situated in alpha helices, beta strands, and random coil regions. Significant difference (*P* < 0.05) is shown by asterisk.

In Figure 4(a), we placed average usage of lysine around Glu residues involved in Mn^2+^ binding and those Glu residues which are not involved in binding. The difference is significant only for Glu residues in alpha helices: the usage of Lys around Glu residues which are not involved in binding is about 3 times higher than that around Glu residues binding Mn^2+^. It means that the presence of Lys near Glu residue in alpha helix strongly decreases its ability to participate in Mn^2+^ binding. Once again, we have to highlight that lysine is known to be helix former, as well as glutamic acid [16]. So, they should be situated near each other in helices at a high probability. Some parts of those pairs should be involved in helix stabilization by the way of polar interactions or even salt bridges formation. Probably, those interactions do not allow oxygen atoms from side chains of Glu to participate in Mn^2+^ binding. With the growth of GC-content, the usage of lysine in helices decreases, while the usage of glutamic acid does not decrease (or does not decrease as steeply as the usage of lysine) [11]. That is why some glutamic acid residues from alpha helices become available for Mn^2+^ binding under the influence of mutational GC-pressure.

Arginine is encoded by six codons. Four of those codons are GC-rich (CGX). The usage of arginine in three groups of bacterial proteins used in this study is growing with the increase of genomic G + C (4.12 ± 0.46%; 5.46 ± 0.41%; 7.25 ± 0.54%). Even though both lysine and arginine possess positively charged side chains, arginine is not underrepresented in helices around glutamic acid residues (see Figure 4(b)). That is why the increase of arginine usage with the growth of GC-content does not prevent Mn^2+^ binding by glutamic acid residues situated in helices.

### 3.7. Mn^2+^ Binding Amino Acid Residues Are Overrepresented in Such Motifs of Supersecondary Structure as B-BCH-H and B-BCB-B

**Figure 5:**
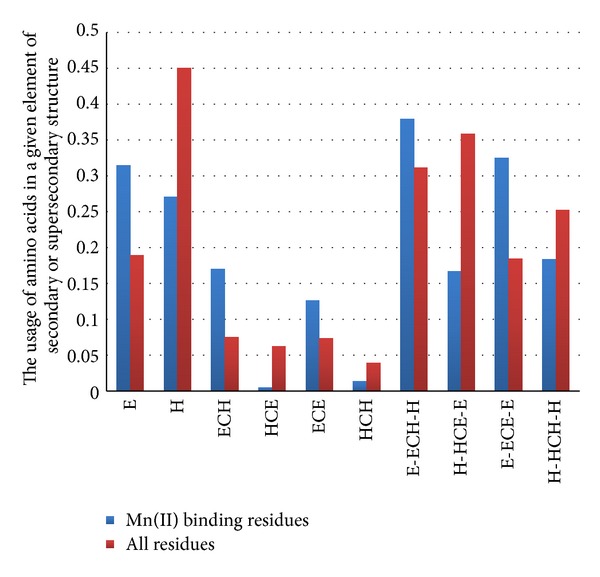
The usage of amino acid residues coordinating Mn^2+^ ions in different types of secondary and supersecondary elements in comparison with the usage of all amino acid residues in subsequent elements. All the differences are significant (*P* < 0.05).

In Figure 5, one can see that the usage of amino acids in beta strands is 1.66 times higher among Mn^2+^ binding residues than among all the residues from the studied proteins (*P* < 0.001). In contrast, the usage of amino acids in alpha helices is 1.66 times lower among Mn^2+^ binding residues than among all the residues (*P* < 0.001).

Regions of coil between beta strand and alpha helix (BCH) contain much more amino acid residues coordinating Mn^2+^ than regions of coil between alpha helix and beta strand (HCB) (see Figure 5). The usage of amino acids situated in the BCH region is 2.3 times higher in the set of residues coordinating manganese cations relatively to the whole set (*P* < 0.001).

Amino acids binding Mn^2+^ ions are significantly overrepresented in regions of coil between two beta strands (BCB) and significantly underrepresented in regions between two alpha helices (HCH) (see Figure 5).

To complete the study, we compared usages of amino acids in the long sequences forming certain supersecondary structure motifs in the set of Mn^2+^ coordinating residues and in the complete set of them. According to our results, Mn^2+^ ions avoid such supersecondary structure motifs as H-HCB-B and H-HCH-H (see Figure 5). Such motifs as B-BCH-H and B-BCB-B are quite suitable for Mn^2+^ coordination (see Figure 5). One may say that both alpha helix and beta strand may be situated after the “beta strand-major binder-random coil” motif.

### 3.8. Comparison between Apo and Holo Forms of Mn^2+^ Binding Proteins

There were 61 amino acid residues coordinating Mn^2+^ ions in 15 proteins for which apo forms with 100% identical amino acid sequences have been found. Interestingly, 46.7% (7 from 15) of apo forms do not differ from holo forms in secondary structures around Mn^2+^ coordinating amino acids. Moreover, there are no differences in secondary structure elements distribution around 72.1% (44 from 61) of those Mn^2+^ coordinating amino acids.

Around 13.1% (8 from 61) of Mn^2+^ binding amino acids beta strands are shorter in holo forms than in apo forms. The difference between their lengths varies from 1 to 3 residues. It means that sometimes coordination of Mn^2+^ ions may lead to the beta strand to coil transition. On the other hand, there are two cases (3.3%) when beta strand is a little bit longer in holo form than in apo form.

In two cases, the difference between structures of apo and holo forms is associated with the fact that some residues situated around Mn^2+^ coordinating amino acids were not located in crystallographic experiment. Other differences are caused by alpha helix to 3/10 helix transition (3.3%), coil to 3/10 helix transition (3.3%), and 3/10 helix to coil transition (1.6%). On one hand, Mn^2+^ ions (as well as other ions) may cause some changes in secondary structures around their binding sites: if atoms from amino acid residues form coordination bonds with cation, they cannot participate anymore in some previously existing interactions stabilizing secondary structure elements. On the other hand, one may find some minor differences between 3D structures of two 100% identical proteins without any ligands or with the same set of ligands. Anyway, differences in secondary structures between apo and holo forms for Mn^2+^ binding proteins are rare and minor.

## 4. Discussion

In our opinion, such supersecondary structural motif as B-BCH-H is suitable for Mn^2+^ coordination because of some specific amino acid propensities. At first, N-termini of helices are enriched by negatively charged amino acid residues: aspartic and glutamic acids [18]. At second, BCH regions demonstrate decreased usage of positively charged amino acids: lysine and arginine [19]. Because of these reasons, B-BCH-H motifs should frequently carry a total negative charge which should attract positively charged cations, such as Mn^2+^ [19]. In contrast, H-HCB-B motifs should usually carry a total positive charge: both C-termini of helices and HCB regions are enriched by lysine and arginine [19].

There should be certain features of B-BCB-B motifs of supersecondary structure which make them suitable for Mn^2+^ binding. Indeed, BCB regions are enriched by such major Mn^2+^ binder, as Asp [19]. Those regions of coil are flexible because of the enrichment by glycine residues [19]. This feature should play some role in the successful coordination of ions. Moreover, BCB regions are more hydrophilic than HCH ones [19].

It is known that the binding of metal ions may induce changes in secondary structure of proteins. For example, it was shown that Ca^2+^ ions are able to promote intermolecular beta-sheet formation by human prion protein (90-231 fragment)* in vitro* [20]. Aggregation of another amyloidogenic protein (alpha-synuclein involved in Parkinson disease pathogenesis) was shown to be accelerated by Cu^2+^ binding [21]. Alzheimer's beta amyloid peptides in fibril form were shown to be able to bind Cu^2+^ ions [22]. Calcitonin was shown to form aggregates in the presence of Cu^2+^, Zn^2+^, and Al^3+^ ions [23]. So, it is important to discuss here the question on causes and consequences.

We showed that there is usually beta strand in the N-terminal direction from the residue binding Mn^2+^. There are just a few apo structures available for Mn^2+^ coordinating bacterial proteins. Even though changes induced by Mn^2+^ binding are rare and minor in our data set, stability of beta strands found in N-terminal direction of coordinating residues has to be checked bioinformatically. According to the data from Tables 2–4, beta strand formers (Val, Ile, Phe, and Leu) are overrepresented in certain positions in the N-terminal direction from three major binders (Asp, His, and Glu). So, beta strands near Mn^2+^ binding sites should be formed by strong beta formers. It means that most of those beta strands are quite predictable: they should exist in apo forms of the proteins and they should not be destroyed after the binding of Mn^2+^ ion.

According to the propensity scale [14], certain hydrophobic (WOOOO; OWOOO; OOOWO; OOOOW; OOOOO) and amphiphilic (WOWOW; WOWOO; OOWOW; OWOWO) pentapeptides are overrepresented in beta strands. We calculated total usage of those sheet-like pentapeptides in “−5–−1” and “−4–0” positions for amino acid residues involved in Mn^2+^ binding in case if there were beta strands in “−3” and “−2” positions, respectively.

**Figure 6:**
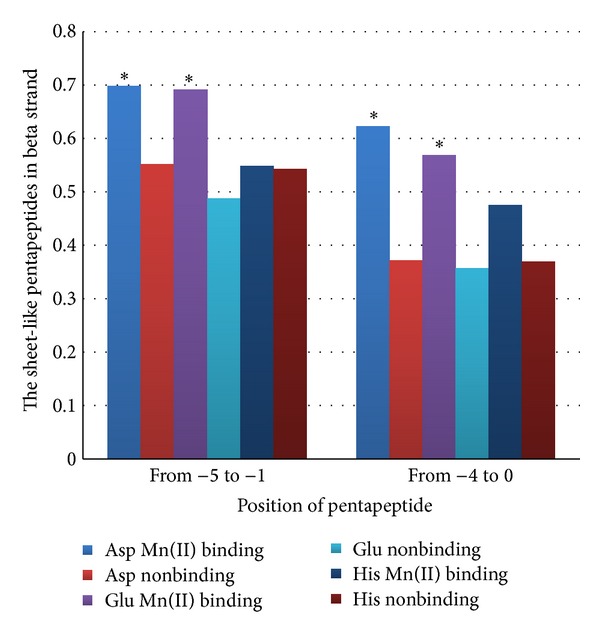
The usage of sheet-like pentapeptides in beta strands situated in “−5–−1” and “−4–0” positions before Asp, Glu, and His residues involved and not involved in Mn^+2^ binding. Significant differences (*P* < 0.05) are designated by asterisks.

As one can see in Figure 6, the percentage of sheet-like pentapeptides in “−5–−1” positions from the Asp involved in Mn^2+^ binding is significantly higher than that percentage for Asp which is not involved in metal ion coordination (69.84% versus 55.13%; *P* < 0.01). The difference for “−4–0” pentapeptides is even higher (62.28% versus 37.12%; *P* < 0.001). Beta strands near the aspartic acid residues from Mn^2+^ binding sites are formed from sheet-like pentapeptides even more frequently than beta strands near Asp residues which are not involved in binding. So, the kind of secondary structural site for Mn^2+^ binding described in the present work (“beta strand-major binder-random coil”) should be stable. Beta strands from those sites should not be formed or destroyed due to the Mn^2+^ binding.

The same tendency is characteristic for glutamic acid residues binding Mn^2+^. Pentapeptides in “−5–−1” positions are sheet-like in 69.09% beta strands situated near the Glu binding Mn^2+^ and just 48.77% of them are sheet-like in case if Glu is not involved in binding (*P* < 0.01). For “−4–0” pentapeptides, the difference is somewhat higher (56.90% versus 35.68%; *P* < 0.01).

Beta strands situated near histidine residues involved in Mn^2+^ binding contain approximately the same percentage of sheet-like pentapeptides as those situated near histidine residues which are not involved in binding (see Figure 6).

In general, we can state that beta strands in “beta strand-major binder-random coil” secondary structural motifs for Mn^2+^ binding are stable enough since both amino acid residues known as strong beta formers and sheet-like pentapeptides are overrepresented in them.

It is known that the “two-histidines-one-carboxylate” binding motif is a widely represented first coordination sphere motif present in the active site of a variety of metalloenzymes [24]. Since histidine and two amino acid residues with carboxyl groups in their side chains are the major binders of Mn^2+^, this motif should be present in our data set as well. However, there are just 11 from 215 (5.12%) Mn^2+^ binding sites which consist of two histidines and a single glutamic or aspartic acid. The percentage of sites with three amino acid residues (28.37%) is lower than the percentage of sites with four amino acid residues (36.74%). There also may be five (5.12%), two (17.67%), or even a single amino acid residue (12.09%) in a binding site. Cases when there is only a single atom from the protein participating in Mn^2+^ coordination can be explained by the fact that there are also several atoms from another ligand bound to that protein interacting with Mn^2+^. Oxygen atoms from water molecules are also frequently described as those participating in Mn^2+^ coordination.

Since there are usually four or three amino acid residues in Mn^2+^ binding site, it is very interesting to estimate the percentage of “type I” sites containing at least one amino acid residue with characteristic beta strand in the N-direction. This percentage is equal to 77.78% for proteins from GC-poor bacteria; 80.00% for proteins from bacteria with average genomic GC-content; and 74.63% for proteins from GC-rich bacteria. The differences between those values are insignificant.

Theoretically, existence of at least one “beta strand-major binder-random coil” secondary structural motif may be important for successful Mn^2+^ binding. In proteins encoded by GC-rich genes, the percentage of binding Glu residues situated in alpha helices increased significantly, while most of those residues bind the ion together with at least one amino acid from characteristic “beta strand-major binder-random coil” motif. It is likely that amino acid residues in that characteristic secondary structural motif are “active” Mn^2+^ binders, while all the other atoms are included in coordination sphere just because they are situated near that “active” binder. On the other hand, 20–25% of Mn^2+^ ions were bound by proteins without involvement of the characteristic “beta strand-major binder-random coil” structural motif. Most of those proteins coordinate Mn^2+^ ions by “type II” sites which are made from Glu residues included in helices with low Lys usage.

## 5. Conclusions

In this work, we used a new bioinformatical approach to study the preferences in secondary structure motifs for metal coordinating amino acid residues microenvironment. Three sets of PDB files have been collected in respect of GC-content of genes encoding the proteins with determined three-dimensional structures. With the help of this approach, one will be able not only to test whether the data are reproducible in three different sets, but to find out previously unknown consequences of symmetric mutational pressure.

In this particular study, we showed that beta strand is often situated before the amino acid residue participating in Mn2+ ion coordination, region of coil is usually situated after the interacting residue, that region of coil may connect above-mentioned beta strand with either another beta strand or alpha helix. This information is useful for future development of an algorithm for Mn(II) binding sites prediction. Moreover, we showed that mutational GC-pressure leads to the more frequent involvement of glutamic acid residues situated in alpha helices into the Mn^2+^ coordination.

## Supplementary Material

Supplementary material includes three MS Excel files.
1. "PDB identifiers.xlsx" file includes complete list of PDB identifiers of analyzed proteins divided into three groups (for proteins from bacteria with low, average and high genomic GC-content).
2. "Mn(II) binding sites.xlsx" file includes three sets of sequences 11 amino acids in length with Mn(II) binding residues in their central positions, as well as description of secondary structure elements for those sequences.
3. "D, H and E residues non-binding Mn(II).xlsx" contains three sets of sequences 11 amino acids in length with D, H and E residues which are not involved in Mn(II) binding in their central positions together with description of their secondary structure elements.

## Figures and Tables

**Table 1:** The most common interacting residues in Mn^2+^ binding sites.

Amino acid	GC-poor bacteria		Bacteria with average genomic G + C		GC-rich bacteria	
	% among amino acid residues from Mn^2+^ binding sites	% of amino acid residues involved in binding of Mn^2+^ from the total amino acid usage	% among amino acid residues from Mn^2+^ binding sites	% of amino acid residues involved in binding of Mn^2+^ from the total amino acid usage	% among amino acid residues from Mn^2+^ binding sites	% of amino acid residues involved in binding of Mn^2+^ from the total amino acid usage
Asp	32.92	6.68	33.47	6.73	36.08	7.06
Glu	19.25	2.96	22.18	3.47	22.68	4.11
His	32.92	17.10	27.02	13.24	34.54	14.86

**Table 2:** Probabilities of amino acids and secondary structure elements occurrence near the aspartic acid binding Mn^+2^. Significantly overrepresented amino acids and secondary structure elements are written in **bold** font; significantly underrepresented ones are written in *italic* font.

	Position										
	−5	−4	−3	−2	−1	0	+1	+2	+3	+4	+5
Secondary structure elements											
Alpha helix	*0.1796 *	*0.2099 *	*0.2427 *	*0.2757 *	*0.3284 *	*0.3450 *	*0.3750 *	*0.3923 *	*0.4062 *	*0.4153 *	*0.4229 *
Helix 3/10	*0.1435 *	*0.1510 *	*0.1379 *	*0.1846 *	*0.1902 *	*0.3374 *	0.3890	0.5680	0.5579	0.5497	0.5340
Beta strand	**0.7168**	**0.7501**	**0.7617**	**0.7797**	**0.7586**	**0.7487**	**0.6100**	0.5517	0.5250	*0.4016 *	*0.4096 *
Random coil	0.5246	*0.4327 *	*0.4106 *	*0.4193 *	0.4908	0.5285	**0.5748**	**0.5660**	**0.5771**	**0.6264**	**0.6217**

Amino acid residues											
Gly	**0.6370**	0.5558	*0.3270 *	0.4731	**0.6460**		0.5860	0.4783	**0.6672**	0.4998	0.5666
Ala	0.4842	*0.3034 *	0.4950	*0.3750 *	0.4514		0.5373	*0.3277 *	0.5514	*0.3597 *	0.5503
Arg	*0.2299 *	0.3577	*0.1282 *	0.4743	*0.1549 *		*0.1403 *	0.4793	*0.1268 *	0.5027	0.5420
Pro	0.5154	0.3658	*0.1608 *	0.3739	*0.2431 *		*0.2746 *	*0.2586 *	0.4131	**0.6845**	0.5039
Asp	0.5393	0.4436	*0.3350 *	**0.6726**	0.5070		0.4226	**0.6602**	0.5108	0.6133	0.5779
Glu	*0.2582 *	*0.3646 *	*0.1760 *	*0.0657 *	0.4343		0.4201	*0.3424 *	0.4847	0.4610	0.4299
Ser	0.4719	0.5163	0.4048	0.4725	**0.6910**		0.6380	0.5689	0.5003	0.5770	0.6512
Thr	0.3882	*0.1855 *	0.4723	0.6461	0.5266		**0.6485**	0.4065	0.5931	0.5352	0.4776
His	0.4805	0.6318	0.6570	**0.7083**	*0.1879 *		**0.7599**	**0.7252**	**0.7558**	**0.6928**	0.5135
Gln	0.4885	*0.3112 *	*0.3250 *	0.4054	0.3304		0.5678	0.3378	0.5758	0.4960	0.5348
Leu	**0.6035**	**0.6947**	**0.6182**	0.4934	0.5553		0.4201	0.5345	0.5134	0.4761	0.4477
Val	**0.6213**	0.5765	**0.7197**	0.4899	**0.6191**		0.5851	0.4018	0.4680	0.4045	0.4777
Cys	0.6184	0.5520	0.5529	0.4680	0.7590		0.4053	0.5605	0.5840	0.3918	0.3335
Trp	*0.0000 *	*0.0000 *	0.4445	0.4243	0.5059		0.4874	0.7362	*0.0000 *	0.2677	0.6925
Phe	*0.2463 *	0.4276	0.5089	0.4954	0.6110		0.5702	0.5567	0.4872	0.4516	0.4760
Tyr	0.4526	0.5446	0.5939	0.5095	0.3509		0.3762	0.3193	0.5080	0.4889	0.3463
Met	0.5173	0.3223	0.6056	0.6057	0.4699		0.5728	0.5943	0.2946	0.6626	0.4124
Ile	0.6001	0.6224	**0.7370**	0.5852	0.4886		0.4826	0.4942	0.4968	0.4914	*0.3347 *
Asn	0.4551	0.5931	*0.0000 *	0.5943	0.3750		0.3671	**0.6858**	0.4067	0.4742	0.4787
Lys	0.3930	0.3637	*0.2196 *	*0.2408 *	*0.0000 *		*0.1701 *	0.5178	*0.1870 *	*0.2516 *	0.4428

**Table 3:** Probabilities of amino acids and secondary structure elements occurrence near the histidine binding Mn^+2^. Significantly overrepresented amino acids and secondary structure elements are written in **bold** font; significantly underrepresented ones are written in *italic* font.

	Position										
	−5	−4	−3	−2	−1	0	+1	+2	+3	+4	+5
Secondary structure											
Alpha helix	*0.3529 *	*0.3658 *	*0.3722 *	*0.3875 *	*0.3926 *	*0.3743 *	*0.3520 *	*0.4060 *	*0.4216 *	*0.4283 *	*0.4377 *
Helix 3/10	*0.1925 *	*0.1996 *	*0.1087 *	0.3760	0.4942	0.6047	0.6233	0.5132	0.5301	0.5028	0.5619
Beta strand	**0.6909**	**0.7072**	**0.6777**	**0.6241**	**0.6109**	**0.6085**	0.5758	0.4659	0.4537	0.4593	0.5050
Random coil	0.4714	*0.4269 *	0.4756	0.5203	0.5102	0.5062	0.5415	**0.5858**	**0.5881**	**0.5867**	**0.5562**

Amino acid residues											
Gly	0.6023	0.5387	**0.6237**	0.4542	0.6047		0.5111	0.5110	0.4700	0.5090	0.5105
Ala	0.4429	0.4286	*0.3014 *	0.6146	*0.3698 *		**0.6546**	0.3897	0.4648	0.4391	*0.3511 *
Arg	0.5318	0.4737	0.4076	*0.2917 *	0.4558		*0.3026 *	0.4410	0.4804	0.3694	0.5197
Pro	0.4229	0.4390	0.5047	*0.2392 *	0.4987		*0.3511 *	0.3794	0.5078	0.6874	0.5455
Asp	0.3510	0.6344	0.5209	0.6087	**0.6750**		*0.2911 *	0.5713	0.4739	0.4474	0.5105
Glu	0.3804	0.4838	0.5212	0.5326	0.3849		0.4055	0.3864	0.5007	0.4144	**0.6324**
Ser	0.4728	0.5826	0.4980	0.5494	0.5863		**0.7419**	*0.0978 *	0.5219	0.5802	0.5573
Thr	0.4240	0.5182	0.5166	0.5641	0.4518		0.5387	**0.6706**	0.4724	0.3971	**0.6449**
His	0.5489	0.5257	0.5404	**0.7368**	0.4190		0.6764	**0.8082**	*0.2810 *	0.6637	0.5514
Gln	0.5220	*0.2021 *	0.4725	0.3245	*0.1381 *		0.3898	0.5475	0.5442	0.4031	0.5182
Leu	0.4046	0.5090	0.5548	0.4323	0.5618		0.4203	0.4750	0.4355	0.4289	0.4841
Val	0.5500	0.4548	0.6161	0.4453	*0.3470 *		*0.2674 *	*0.3332 *	0.3967	*0.2995 *	0.4563
Cys	0.8949	0.6196	*0.0000 *	0.6837	0.5906		0.2911	0.6570	0.6209	0.6568	0.5891
Trp	0.3405	0.4872	0.2416	0.7546	0.7121		0.7853	0.6885	0.6417	**0.7782**	0.2906
Phe	0.4969	0.3161	0.4387	0.4013	0.3441		0.4560	0.4380	**0.7076**	0.4738	*0.1153 *
Tyr	0.6653	0.3878	0.4100	0.5355	0.5442		0.5640	0.6372	0.6323	0.6707	*0.2106 *
Met	0.5390	0.5246	**0.7035**	0.5043	0.5459		0.4721	*0.2583 *	0.6417	0.3647	0.3892
Ile	**0.6665**	**0.6516**	0.4374	*0.2859 *	0.5639		0.5630	0.5199	0.4174	0.4135	0.5182
Asn	0.5579	*0.2262 *	0.4300	0.5439	0.6292		0.3194	0.5999	0.4114	**0.7416**	0.4828
Lys	0.3661	0.4821	*0.0886 *	*0.1288 *	*0.3034 *		0.3575	*0.1932 *	0.5771	*0.2601 *	0.5197

**Table 4:** Probabilities of amino acids and secondary structure elements occurrence near the glutamic acid binding Mn^+2^. Significantly overrepresented amino acids and secondary structure elements are written in **bold** font; significantly underrepresented ones are written in *italic* font.

	Position										
	−5	−4	−3	−2	−1	0	+1	+2	+3	+4	+5
Secondary structure											
Alpha helix	*0.4389 *	*0.4369 *	*0.4184 *	*0.4169 *	*0.4132 *	*0.4239 *	*0.4369 *	*0.4466 *	0.4658	0.4784	0.4824
Helix 3/10	0.3244	*0.1428 *	*0.1397 *	*0.1206 *	*0.2046 *	*0.2120 *	*0.2941 *	0.3227	0.4288	0.4298	0.4209
Beta strand	0.5667	**0.7125**	**0.7486**	**0.7876**	**0.7906**	**0.7422**	**0.6879**	**0.6441**	0.5720	0.5235	0.4387
Random coil	0.5514	*0.4260 *	*0.4037 *	*0.3634 *	*0.3782 *	0.4596	0.5024	0.5168	0.5190	0.5276	**0.5635**

Amino acid residues											
Gly	0.5211	0.6336	0.4237	0.5153	0.4063		0.5083	0.5928	0.5402	0.5435	0.4032
Ala	0.3723	0.4991	0.5324	0.5452	0.4904		0.5185	0.5102	0.5357	0.3980	0.3706
Arg	0.5484	0.3906	*0.1075 *	0.6085	*0.1092 *		0.4305	0.5382	*0.1652 *	0.6204	0.4530
Pro	*0.2502 *	0.5342	0.5540	*0.1581 *	*0.1908 *		0.5971	0.3686	0.5569	*0.1365 *	0.5218
Asp	0.5290	0.4307	0.3942	0.6160	0.5934		0.3986	0.6196	0.3634	0.5335	0.5604
Glu	0.4256	0.4299	0.3910	0.3654	0.5080		0.4157	*0.3132 *	0.4221	0.4578	0.5145
Ser	0.4331	0.4726	*0.3015 *	0.3992	0.5043		0.5340	0.4678	0.5944	0.5238	0.1456
Thr	0.4928	0.6066	0.5354	0.5818	0.5080		0.5094	0.4405	0.4055	0.6249	0.6619
His	0.6939	0.2517	0.5485	0.6433	0.5667		0.6924	0.6965	**0.8356**	0.5716	0.6361
Gln	0.3413	*0.2860 *	0.3184	0.5547	0.3351		0.4316	0.4573	0.3449	0.4392	0.6356
Leu	0.4900	*0.3047 *	0.5152	0.4727	0.6081		0.4881	*0.3579 *	0.4890	0.4532	0.3911
Val	0.4343	0.5487	**0.6632**	*0.3459 *	0.5596		**0.6440**	*0.2802 *	0.4865	0.5228	0.4701
Cys	*0.0000 *	*0.0000 *	0.7130	0.4774	0.7331		*0.0000 *	0.6770	0.5011	0.4911	0.4146
Trp	0.7566	0.7077	0.3942	*0.0000 *	*0.0000 *		0.8008	*0.0000 *	0.4296	*0.0000 *	0.5235
Phe	0.6538	0.6079	**0.7460**	0.6494	0.6041		0.3868	0.5892	0.5105	0.5135	0.5668
Tyr	0.5313	0.5891	0.4059	0.3326	0.4239		0.4810	0.3842	0.6738	0.5359	0.5808
Met	0.7144	0.4412	0.2897	0.6551	0.6262		0.4713	0.4759	0.5786	0.6281	0.6272
Ile	0.5946	0.6594	0.5899	0.4762	**0.6830**		0.4488	0.5580	0.5384	0.5150	0.5065
Asn	*0.1730 *	0.5097	0.6126	0.4046	0.3917		0.4976	0.6646	0.4686	0.4375	0.6089
Lys	0.3916	*0.3233 *	*0.3320 *	0.3510	*0.2028 *		*0.1066 *	0.5681	*0.2369 *	0.4770	0.4492

## Abbreviations

G + C, GC-content: The usage of guanine and cytosine in a gene or genome
Xaa: Any amino acid
W: Any hydrophilic amino acid (Arg; Lys; His; Asp; Glu; Asn; Gln; Ser; Thr)
O: Any hydrophobic amino acid (Ala; Gly; Pro; Val; Leu; Met; Ile; Tyr; Phe; Cys; Trp)
BCH: Random coil between beta strand and alpha helix
BCB: Random coil between two beta strands
HCB: Random coil between alpha helix and beta strand
HCH: Random coil between two alpha helices
B-BCH-H: Supersecondary structural motif which includes beta strand, coil, and alpha helix (from N- to C-terminus)
B-BCB-B: Supersecondary structural motif which includes beta strand, coil, and beta strand (from N- to C-terminus)
H-HCB-B: Supersecondary structural motif which includes alpha helix, coil, and beta strand (from N- to C-terminus)
H-HCH-H: Supersecondary structural motif which includes alpha helix, coil, and alpha helix (from N- to C-terminus).

## References

[1] Zheng H, Chruszcz M, Lasota P, Lebioda L, Minor W (2008). Data mining of metal ion environments present in protein structures. *Journal of Inorganic Biochemistry*.

[2] Brylinski M, Skolnick J (2011). FINDSITE-metal: integrating evolutionary information and machine learning for structure-based metal-binding site prediction at the proteome level. *Proteins*.

[3] Passerini A, Lippi M, Frasconi P (2011). MetalDetector v2.0: predicting the geometry of metal binding sites from protein sequence. *Nucleic Acids Research*.

[4] Schymkowitz JWH, Rousseau F, Martins IC, Ferkinghoff-Borg J, Stricher F, Serrano L (2005). Prediction of water and metal binding sites and their affinities by using the Fold-X force field. *Proceedings of the National Academy of Sciences of the United States of America*.

[5] Seebeck B, Reulecke I, Kämper A, Rarey M (2008). Modeling of metal interaction geometries for protein-ligand docking. *Proteins*.

[6] Kumar M, Ahmad S, Ahmad E, Saifi MA, Khan RH (2012). In silico prediction and analysis of Caenorhabditis EF-hand containing proteins. *PLoS ONE*.

[7] Kim H-W, Kataoka M, Ishikawa K (2012). Atomic resolution of the crystal structure of the hyperthermophilic family 12 endocellulase and stabilizing role of the DxDxDG calcium-binding motif in Pyrococcus furiosus. *FEBS Letters*.

[8] Rigden DJ, Galperin MY (2004). The DxDxDG motif for calcium binding: multiple structural contexts and implications for evolution. *Journal of Molecular Biology*.

[9] Sueoka N (1988). Directional mutation pressure and neutral molecular evolution. *Proceedings of the National Academy of Sciences of the United States of America*.

[10] Singer GAC, Hickey DA (2000). Nucleotide bias causes a genomewide bias in the amino acid composition of proteins. *Molecular Biology and Evolution*.

[11] Khrustalev VV, Barkovsky EV (2011). Percent of highly immunogenic amino acid residues forming B-cell epitopes is higher in homologous proteins encoded by GC-rich genes. *Journal of Theoretical Biology*.

[12] Khrustalev VV, Barkovsky EV (2010). Study of completed archaeal genomes and proteomes: hypothesis of strong mutational AT pressure existed in Their common Predecessor. *Genomics, Proteomics and Bioinformatics*.

[13] Banerjee T, Gupta SK, Ghosh TC (2005). Role of mutational bias and natural selection on genome-wide nucleotide bias in prokaryotic organisms. *BioSystems*.

[14] Khrustalev VV, Barkovsky EV (2012). Stabilization of secondary structure elements by specific combinations of hydrophilic and hydrophobic amino acid residues is more important for proteins encoded by GC-poor genes. *Biochimie*.

[15] Eisenberg D, Schwarz E, Komaromy M, Wall R (1984). Analysis of membrane and surface protein sequences with the hydrophobic moment plot. *Journal of Molecular Biology*.

[16] Chou PY, Fasman GD (1978). Prediction of the secondary structure of proteins from their amino acid sequence. *Advances in Enzymology and Related Areas of Molecular Biology*.

[17] Yáñez M, Gil-Longo J, Campos-Toimil (2012). Calcium binding proteins. *Advances in Experimental Medicine and Biology*.

[18] Aurora R, Rose GD (1998). Helix capping. *Protein Science*.

[19] Khrustalev VV, Khrustaleva TA, Barkovsky EV (2013). Random coil structures in bacterial proteins. Relationships of their amino acid compositions to flanking structures and corresponding genic base compositions. *Biochimie*.

[20] Sorrentino S, Bucciarelli T, Corsaro A, Tosatto A, Thellung S (2012). Calcium binding promotes prion protein fragment 90–231 conformational change toward a membrane destabilizing and cytotoxic structure. *PLoS ONE*.

[21] Rose F, Hodak M, Bernholc J (2011). Mechanism of copper(II)-induced misfolding of Parkinson’s disease protein. *Scientific Reports*.

[22] Parthasarathy S, Long F, Miller Y (2011). Molecular-level examination of Cu^2+^ binding structure for amyloid fibrils of 40-residue alzheimer’s *β* by solid-state NMR spectroscopy. *Journal of the American Chemical Society*.

[23] Rastogi N, Mitra K, Kumar D, Roy R (2012). Metal ions as cofactors for aggregation of therapeutic peptide salmon calcitonin. *Inorganic Chemistry*.

[24] Amrein B, Schmid M, Collet G (2012). Identification of two-histidines one-carboxylate binding motifs in proteins amenable to facial coordination to metals. *Metallomics*.

